# Uncovering the Pharmacological Mechanism of* Chaibei Zhixian* Decoction on Epilepsy by Network Pharmacology Analysis

**DOI:** 10.1155/2019/3104741

**Published:** 2019-05-12

**Authors:** Jian Zhang, Chenglong Zheng, Siyuan Yuan, Xiaoke Dong, Le Wang, Yong Wang, Wei Wang, Kuo Gao, Jinmin Liu

**Affiliations:** ^1^School of Traditional Chinese Medicine, Beijing University of Chinese Medicine, Beijing 100029, China; ^2^Beijing Gulou Hospital of Traditional Chinese Medicine, Beijing, 10009, China; ^3^Dongfang Hospital, Beijing University of Chinese Medicine, Beijing, 100078, China; ^4^School of Life Science, Beijing University of Chinese Medicine, Beijing 100029, China

## Abstract

**Objective:**

Epilepsy is a neuronal disorder that is characterized by epileptic seizures and linked with abnormal neural functioning in the brain. Traditional Chinese medicine (TCM) formula* Chaibei Zhixian* decoction (CZD) has been widely used for epilepsy in China while the pharmacological mechanisms are still unclear. In the present study, systematic and comprehensive network pharmacology was utilized for the first time to reveal the potential pharmacological mechanisms of CZD on epilepsy.

**Methods:**

Traditional Chinese Medicine Systems Pharmacology (TCMSP) database and analysis platform was utilized for the development of an ingredients-targets database. After identifying epileptic targets of CZD, their interaction with other proteins was estimated based on protein-protein interaction network created from STITCH and gene ontology (GO) enrichment analysis utilizing Cytoscape-ClueGO plugin.

**Results:**

CZD formula was found to have 643 chemical ingredients, and the potential protein targets of these ingredients were 5230, as retrieved from TCMSP database. Twenty-six protein targets were found to be associated with epilepsy. Thirteen hub genes were regulated by CZD in epilepsy, including estradiol, ESR1, ESR2, SRC, CTNNB1, EP300, MAPK1, MAPK3, SP1, BRCA1, NCOA3, CHRM1, and GSK3B. The results of GO terms analysis showed that 8 GO terms were recovered in the form of 3 clusters, including negative regulation of protein kinase B signaling, positive regulation of interleukin-1 production, and microvillus assembly.

**Conclusions:**

Network pharmacology approach provides better understanding of the underlying pharmacological mechanisms of CZD on epilepsy. Estradiol, ESR1, ESR2, CTNNB1, EP300, MAPK1, MAPK3, BRCA1, and GSK3B are likely to be important molecules regulated by CZD in treatment of epilepsy. Negative regulation of protein kinase B signaling may play vital roles in the treatment of epilepsy by CZD.

## 1. Introduction

Epilepsy is a complex disorder involving neurological alterations that lead to the pathological development of recurrent seizures [1, 2]. Epilepsy affects millions of people worldwide and approximately one-third of patients suffer from cognitive impairment, particularly memory disruption [1, 3, 4]. First-line antiepileptic drugs have been given priority in the clinical treatment of epileptic seizures [1]. However, the risk of adverse effects from antiepileptic drugs is considerable and includes potential cognitive and behavioral effects [5]. Therefore, strategies that reduce the side effects of antiepileptic drugs or develop new drugs are urgently needed for epilepsy therapies.

Traditional Chinese medicine (TCM) has a long history in prevention and treatment of epilepsy in China [6, 7].* Chaibei Zhixian* decoction (CZD), composed of Radix Bupleuri, Bulbus Fritillariae Thunbergii, Rhizoma Gastrodiae, Rhizoma Pinelliae, Rhizoma Acori Tatarinowii, Concha Ostreae, and Pheretima in a 4:3:5:3:3:10:2 ratio (Table 1), has been widely used in clinical treatment of epilepsy in China. Clinical study has shown that CZD is safe and effective for intractable epilepsy [8]. In addition, the combination of CZD with first-line antiepileptic drugs could reduce side effects and increase curative effects [9]. Some experimental studies have found CZD to have therapeutic effects on epilepsy by regulating multidrug resistance-associated protein 1, nuclear factor-kappa B, breast cancer resistance protein, and p-glycoprotein [10–13]. These studies all use traditional research method of single-drug, single-target, and single-pathway, but the TCM formula CZD has the characteristics of being multicomponent, multitarget, and multipathway. Thus, a new comprehensive and systematic evaluation of the pharmacological mechanism of CZD on epilepsy is critically needed.

Network pharmacology, including chemoinformatics, bioinformatics, network biology, and pharmacology, is a comprehensive method to uncover the bioactive components and potential mechanisms of TCM formulas from a systemic perspective [14]. In this study, the potential pharmacological mechanisms of CZD on epilepsy have been probed using network pharmacology, drug-target interaction databases, and a biological process analysis.

## 2. Methods

The first step of this study involved the retrieval of CZD constituents and their target proteins in* Homo sapiens*. Then the construction of CZD-target interaction network and its analysis was accomplished by using various GO terms. Finally, to assess the molecular mechanisms of CZD effects in epilepsy, Cytoscape along with its plugin ClueGO was utilized for GO enrichment analysis, followed by the analysis of biological processes.

### 2.1. Chemical Search and Their Target Retrieval

CZD contains Radix Bupleuri, Bulbus Fritillariae Thunbergii, Rhizoma Gastrodiae, Rhizoma Pinelliae, Rhizoma Acori Tatarinowii, Concha Ostreae, and Pheretima (Table 1). The chemical constituents present in these seven sources as well as the protein targets of these chemicals were searched via Traditional Chinese Medicine Systems Pharmacology Database and Analysis Platform (TCMSP, http://5th.tcmspw.com/tcmsp.php) [15]. The duplications in these chemical constituents from various sources and their targets were removed. From the retrieved targets, epilepsy-related targets were screened via Kyoto Encyclopedia of Genes and Genomes (KEGG, http://www.kegg.jp/) and employed for further analysis.

### 2.2. Conversion of Target Proteins into Network and Its Analysis

STITCH 5.0 database (http://stitch.embl.de/) [16] was utilized for analysis of the interaction among the identified protein targets to systematically investigate the mode of action of CZD. STITCH database has been furnished with extensive information regarding protein interactions. The main sources of information about these interactions are genomic model evaluations and high-throughput experimental outcomes. The information about > 9 million proteins from more than 2000 organisms has been added to this database. The mechanism of action of CZD and its essential pharmacodynamic constituents was assessed by developing a protein interaction network. The optional setting for network construction was set as follows: number of interactors = not more than 10; minimum required interaction score = 0.700 [16].

### 2.3. GO Terms Analysis through ClueGO Plugin

After identifying typical biological features of the protein targets, the ingredients of protein interaction network obtained from STITCH were used in ClueGO-based analysis, and GO enrichment analysis was introduced for the segmentation of target genes in a hierarchically arranged manner. ClueGO was utilized as a plugin of Cytoscape 3.4.0 software to construct [17], visualize, and evaluate protein target network and to study the biological pathways [18]. ClueGO analysis was conducted at a level of significance of 0.05. This study assumed that the network was of medium type. In contradiction to the detailed and global networks, medium network establishes GO terms belonging to GO levels 4-8, having a medium number of associated genes, and a medium percentage of uploaded genes found. Furthermore, two-sided hypergeometric test along with Bonferroni correction was used in network analysis. Lastly, organic layout algorithm was employed to visualize the functional network.

## 3. Results

### 3.1. Chemical Search and Their Target Retrieval

TCMSP search resulted in the retrieval of 643 chemical ingredients in the five herbs, Radix Bupleuri, Bulbus Fritillariae Thunbergii, Rhizoma Gastrodiae, Rhizoma Pinelliae, and Rhizoma Acori Tatarinowii, and two animals, Concha Ostreae and Pheretima. The protein targets of these 643 chemical ingredients recovered from TCMSP database were 5230 in number (Supplemental Table 1). Some studies revealed that CZD could be used for treating epilepsy [8–13]. After removing the repeated protein targets, 941 protein targets still remain (Supplemental Table 2). Among them, 26 protein targets of CZD were found to be associated with epilepsy through scanning TCMSP database (Supplemental Table 3), followed by their standardization through UniProt database mapping (http://www.uniprot.org/).

### 3.2. Conversion of Target Proteins into Network and Its Analysis

**Figure 1:**
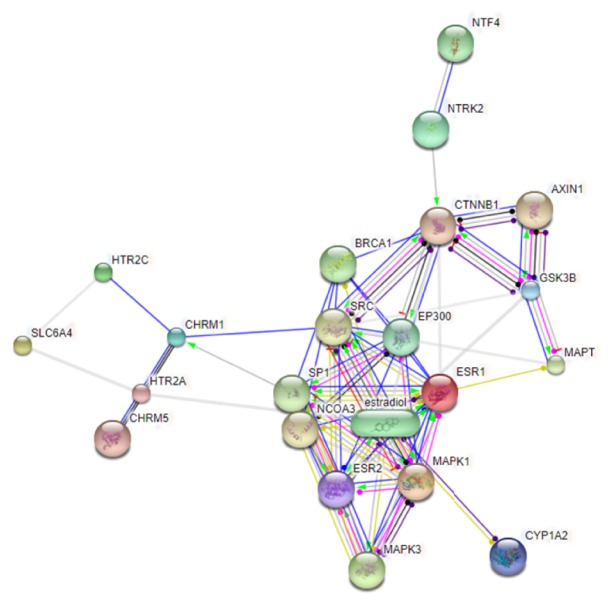
PPIN (action view) showing CZD targets. The colored edges indicate the nature of action, as interpreted here: activation (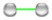), inhibition (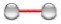), binding (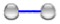), catalysis (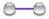), phenotype (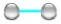), posttranslational modification (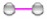), reaction (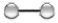), and transcriptional regulation (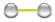). The effects of action are represented by the following symbols: positive (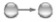), negative (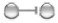), and unspecified (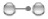). AXIN1: axin 1; NCOA3: nuclear receptor coactivator 3; BRCA1: breast cancer 1, early onset; CHRM1: cholinergic receptor, muscarinic 1; CHRM5: cholinergic receptor, muscarinic 5; ESR1: estrogen receptor 1; CTNNB1: catenin (cadherin-associated protein), beta 1; CYP1A2: cytochrome P450, family 1, subfamily A, polypeptide 2; ESR2: estrogen receptor 2; HTR2A: 5-hydroxytryptamine (serotonin) receptor 2A; GSK3B: glycogen synthase kinase 3 beta; MAPK1: mitogen-activated protein kinase 1; MAPK3: mitogen-activated protein kinase 3; HTR2C: 5-hydroxytryptamine (serotonin); MAPT: microtubule-associated protein tau; SP1: Sp1 transcription factor; receptor 2C; NTF4: neurotrophin 4; EP300: E1A binding protein p300; NTRK2: neurotrophic tyrosine kinase, receptor, type 2; SLC6A4: solute carrier family 6 (neurotransmitter transporter, serotonin), member 4; SRC: v-src sarcoma (Schmidt-Ruppin A-2) viral oncogene homolog (avian).

The systematically selected protein targets with a probabilistic confidence score of 0.700 were plotted as an interaction network (Figure 1) by using STITCH database (accessed in Oct 2018). The number of nodes and edges of this network were 21 and 48, respectively. Out of 21, there were 11 physical and 10 functional interactions. STITCH is a database of known and predicted interactions between chemicals and proteins. The interactions include direct (physical) and indirect (functional) associations; they stem from computational prediction, from knowledge transfer between organisms, and from interactions aggregated from other (primary) databases. The nodes and the edges represent protein/gene targets and their interactions, respectively. In case of random selection of nodes, the expected number of edges of the acquired PPIN was 23. The statistics of PPIN enrichment had a very small p-value (3.56E-06), indicating arbitrary nature of nodes and a significant number of edges.

The average node degree and clustering coefficient are the other important features of PPIN. Degree is a topological parameter that refers to the number of connections between a node and other nodes and can be used to describe the characteristics (particularly centrality) of nodes in the network. Node degree is a quantitative feature of a node that represents the number of linkages of a node in a network. A higher degree means a stronger correlation. The average node degree refers to the mean number of associations of a protein in a PPIN at threshold score, whereas the connectivity degree of PPIN nodes is indicated by the clustering coefficient. An increase in the clustering coefficient results in the increase in network connectivity. The average node degree and clustering coefficient values were 4.57 and 0.57, respectively. A node is termed as hub if it has number of linkage higher than its average node degree. Thirteen hubs have higher node degree than the average node degree, including estrogen receptor 1 (ESR1), estradiol, v-src sarcoma (Schmidt-Ruppin A-2) viral oncogene homolog (avian) (SRC), catenin beta 1 (CTNNB1), E1A binding protein p300 (EP300), estrogen receptor 1 (ESR2), mitogen-activated protein kinase 1 (MAPK1), Sp1 transcription factor (SP1), breast cancer 1, early onset (BRCA1), nuclear receptor coactivator 3 (NCOA3), cholinergic receptor, muscarinic 1 (CHRM1), glycogen synthase kinase 3 beta (GSK3B), and mitogen-activated protein kinase 3 (MAPK3) (Table 2). In addition, the functional proteins except NCOA3 and BRCA1 could be activated by CZD, which, on the other hand, inhibit CTNNB1, AXIN1, SRC, MAPT, SP1, and estradiol (Table 3). All functional proteins, as listed in Table 3, can bind with CZD. Moreover, the catalysis, posttranslational modifications, reactions, and expression are also found to be affected by CZD (Table 3).

### 3.3. GO Terms through ClueGO Plugin

**Figure 2:**
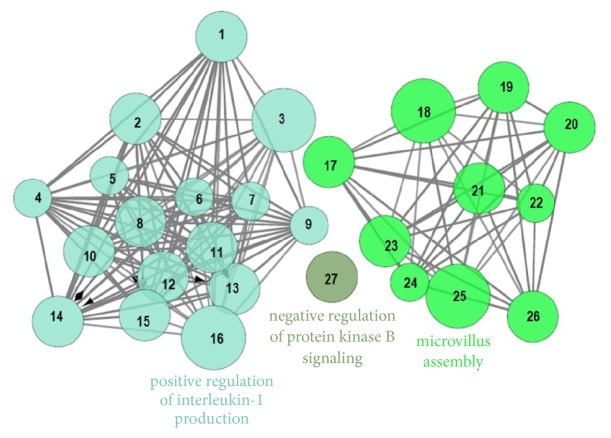
Targets involved in the biological effects. The most significant term in each stack is used to label the respective group. Node size is directly related to the term enrichment significance. The groups of GO terms having similar function are partially overlapped. [1-AIM2, 2-AZU1, 3-CALCA, 4-CARD8, 5-CASP1, 6-CASP5, 7-CCL19, 8-EGR1, 9-GSDMD, 10-HAVCR2, 11-HDAC2, 12-HMGB1, 13-HSPB1, 14-NOD1, 15-SMAD3, 16-TLR4, 17-ATP8B1, 18-EZR, 19-FSCN1, 20-FXYD5, 21-PLD1, 22-PRKCSH, 23-RAP1A, 24-RAPGEF2, 25-RAPGEF6, 26-SLC9A3R1, 27-PHLPP1].

The annotation of biological functions was carried out by using GO terms and ClueGO plugin. This enrichment analysis of CZD targets resulted in the evolution of 8 GO terms which were ordered into 3 subgroups, including negative regulation of protein kinase B signaling, positive regulation of interleukin-1 production, and microvillus assembly (Table 4, Figure 2).

## 4. Discussion

Epilepsy affects millions of people worldwide and approximately one-third of patients suffer from cognitive deficits. Due to the side effects of first-line antiepileptic drugs, more effective treatments are still needed. The TCM formula CZD not only is safe and effective for intractable epilepsy but also reduces side effects and increase curative effects when in combination with first-line antiepileptic drugs. However, the underlying mechanism of CZD on epilepsy is still unclear and remains unrevealed from a systemic point of view. Therefore, we adopted network pharmacology to further explore the mechanisms of CZD on epilepsy in this study. This systematic network pharmacology approach is a combination of various procedures, including retrieval of chemical ingredients of CZD, target search of these chemicals, development of network using these targets, and GO terms analysis. CZD formula was found to have 643 chemical ingredients, and the potential protein targets of these ingredients were 5230. Two aspects aroused our attention: first, 26 protein targets were found to be associated with epilepsy. Some of them are likely to be key molecules in the treatment of epilepsy with CZD. Second, GO terms analysis indicated that negative regulation of protein kinase B signaling, positive regulation of interleukin-1 production, and microvillus assembly have linkage with CZD treatment for epilepsy.

Network pharmacology analysis has shown that 13 hub genes were regulated by CZD in epilepsy, including estradiol, ESR1, ESR2, SRC, CTNNB1, EP300, MAPK1, MAPK3, SP1, BRCA1, NCOA3, CHRM1, and GSK3B. Among them, estradiol, ESR1, ESR2, CTNNB1, EP300, MAPK1, MAPK3, BRCA1, and GSK3B are closely related to epilepsy based on current studies. Thus, they are likely to be the main regulators of CZD in treatment of epilepsy. Estrogens affect neuronal excitability and have neuroprotective effects on seizure-induced hippocampal damage [19, 20]. Several studies have confirmed ESR was associated with epilepsy [21–24]. CTNNB1 has been implicated in epilepsy because of its altered postseizure expression [25, 26]. The dysfunction of CTNNB1-mediated signaling pathways leads to cortical malformation and increased seizure susceptibility [25]. EP300 may serve as potential targets for the treatment of epilepsy based on gene expression profile analysis of brain tissue of patients with epilepsy [27]. MAPK, as an important regulator of synaptic excitability, exerts an influence on epilepsy in animal models as well as human disease [27–30]. Interestingly, the component of CZD, gastrodin, has been reported to attenuate seizures by modulating the MAPK-associated inflammatory responses [31]. Variants in BRCA1-associated protein required for ATM activation-1 cause multifocal seizure syndrome [32, 33]. GSK3B activity protects neuronal networks from hyperactivation in response to epileptogenic stimuli [34]. Given that some main hubs of PPIN are closely related to epilepsy, they are likely to be important molecules regulated by CZD in treatment of epilepsy.

GO terms analysis revealed that negative regulation of protein kinase B signaling, positive regulation of interleukin-1 production, and microvillus assembly have linkage with CZD treatment for epilepsy. Protein kinase B, a serine/threonine-specific protein kinase, is involved in the regulation of binding phospholipids, phosphorylation, and ubiquitination but also modulates a wide array of cellular processes including cell apoptosis, metabolism, and proliferation [35]. Activation of the Akt signaling could alleviate neuronal apoptosis and oxidative stress [36]. The other two GO terms are positive regulation of interleukin-1 production and microvillus assembly. The former term represents a process in which the production of interleukin-1 is positively regulated, while the latter refers to the formation of microvillus. The positive regulation of interleukin-1 production has been found to be linked with three genes (i.e., AZU1, HMGB1, and TLR4). Microvillus assembly is regulated under the effect of three genes such as RAP1A, RAPGEF2, and SLC9A3R1. It has been reported that microvilli-like entities are linked with the inward movement of lethal capsulated neisseria meningitidis into vascular endothelial cells that may affect BBB resulting in seizures [37, 38].

In short, the present study has suggested various modes of CZD action against epilepsy, revealing that CZD profoundly enhances the performance of target genes involved in inhibiting epilepsy. The limitation of this study is that the bioactive components and targets found by network pharmacology analysis are the result of theoretical predictions and they should be verified by experiments. Further study will focus on using animal experiments and clinical trials to verify the hypothesis.

## 5. Conclusion

Network pharmacology analysis provides better understanding of the underlying pharmacological mechanisms of CZD on epilepsy. Our results revealed that estradiol, ESR1, ESR2, CTNNB1, EP300, MAPK1, MAPK3, BRCA1, and GSK3B are likely to be important molecules regulated by CZD in treatment of epilepsy. In addition, negative regulation of protein kinase B signaling may play vital roles in the treatment of epilepsy by CZD.

## Figures and Tables

**Table 1:** Pharmaceutical ingredients of *Chaibei Zhixian* decoction.

Latin name	Species	Family	Part used
Radix Bupleuri	*Bupleurum Chinese *DC. *Bupleurum scorzonerifolium *Willd.	*Umbelliferae*	Roots

Bulbus Fritillariae Thunbergii	*Fritillaria thunbergii *Miq.	*Liliaceae *	Bulbs

Rhizoma Gastrodiae	*Gastrodia elata *Bl.	*Orchidaceae*	Rhizomes

Rhizoma Pinelliae	*Pinellia ternata *(Thunb.) Breit.	*Araceae *	Rhizomes

Rhizoma Acori Tatarinowii	*Acorus tatarinowii *Schott.	*Araceae *	Rhizomes

Concha Ostreae	*Ostrea gigas *Thunb. *Ostrea talienwhanensis *Crosse *Ostrea rivularis *Gould	*Ostreidae*	Concha

Pheretima	*Pheretima aspergillum *(E. Perrier)	*Megascolecidae*	Bodies

The ratio of these herbs was 4:3:5:3:3:10:2

**Table 2:** Node degree of the targets of CZD acquired via STITCH database.

Targets	Node Degree	Targets	Node Degree
ESR1	11	GSK3B	5
Estradiol	12	MAPK3	5
SRC	10	HTR2A	4
CTNNB1	8	MAPT	3
EP300	8	AXIN1	2
ESR2	8	CHRM5	2
MAPK1	8	HTR2C	2
SP1	8	NTRK2	2
BRCA1	7	SLC6A4	2
NCOA3	6	CYP1A2	1
CHRM1	5	NTF4	1

**Table 3:** Nature of action of functional targets of CZD acquired via STITCH.

Functional targets	Activation	Inhibition	Binding	Phenotype	Catalysis	Post-Trans. Mod.	Reaction	Expression	Score
CTNNB1	•	•	•		•	•	•		0.999
AXIN1	•	•	•		•	•	•		0.999
NCOA3			•						0.999
SRC	•	•	•		•	•	•	•	0.999
MAPT	•	•	•			•		•	0.999
SP1	•	•	•			•		•	0.999
BRCA1			•						0.999
NTF4	•		•						0.999
Estradiol	•	•	•		•			•	0.999

**Table 4:** Recovery of GO terms and the associated genes.

GO ID	GO Term	Term *p* Value (*¤*)	Group *p* Value (*¤*)	Associated Genes Found
51898	Negative regulation of protein kinase B signaling	750.0E-6 (4.5E-3)	750.0E-6 (750.0E-6)	PHLPP1, SLC9A3R1
32732	Positive regulation of interleukin-1 production	6.7E-6 (140.0E-6)	64.0E-6 (190.0E-6)	AZU1, HMGB1, TLR4
30033	Microvillus assembly	1.7E-6 (39.0E-6)	420.0E-6 (840.0E-6)	RAP1A, RAPGEF2, SLC9A3R1

^¤^Corrected with Bonferroni step down.

## Data Availability

The data used to support the findings of this study are available from the corresponding author upon request.

## Supplementary Materials

Supplementary 1 Supplemental Table 1: 5230 protein targets from the TCMSP database.

Supplementary 2 Supplemental Table 2: nonrepetitive 941 protein targets from the TCMSP database.

Supplementary 3 Supplemental Table 3: 26 protein targets of CZD associated with epilepsy through scanning TCMSP database.
